# W_x_C-β-SiC Nanocomposite Catalysts Used in Aqueous Phase Hydrogenation of Furfural

**DOI:** 10.3390/molecules22112033

**Published:** 2017-11-22

**Authors:** Jacek Rogowski, Mariusz Andrzejczuk, Joanna Berlowska, Michal Binczarski, Dorota Kregiel, Andrzej Kubiak, Magdalena Modelska, Elzbieta Szubiakiewicz, Andrei Stanishevsky, Jolanta Tomaszewska, Izabela Alina Witonska

**Affiliations:** 1 Institute of General and Ecological Chemistry, Faculty of Chemistry, Lodz University of Technology, Zeromskiego 116, 90-924 Lodz, Poland; jacek.rogowski@p.lodz.pl (J.R.); michalbinczarski@gmail.com (M.B.); magdalena.modelska@dokt.p.lodz.pl (M.M.); elzbieta.szubiakiewicz@p.lodz.pl (E.S.); trukan.trukan@gmail.com (J.T.); 2 Faculty of Materials Science and Engineering, Warsaw University of Technology, Woloska 141, 02-507 Warsaw, Poland; mandrzej@inmat.pw.edu.pl; 3 Institute of Fermentation Technology and Microbiology, Faculty of Food Science and Biotechnology, Lodz University of Technology, Wolczanska 171/173, 90-924 Lodz, Poland; joanna.berlowska@p.lodz.pl (J.B.); dorota.kregiel@p.lodz.pl (D.K.); 4 Department of Semiconductor and Optoelectronic Devices, Faculty of Electrical, Electronic, Computer and Control Engineering, Lodz University of Technology, ul. Wolczanska 211/215, 90-924 Lodz, Poland; andrzej.kubiak@p.lodz.pl; 5 Department of Physics, University of Alabama at Birmingham, 1300 University Blvd., Birmingham, AL 35294, USA; astan@uab.edu

**Keywords:** furfural, nanostructured catalyst, hydrogenation, tetrahydrofurfuryl alcohol, furfuryl alcohol

## Abstract

This study investigates the effects of the addition of tungsten on the structure, phase composition, textural properties and activities of β-SiC-based catalysts in the aqueous phase hydrogenation of furfural. Carbothermal reduction of SiO_2_ in the presence of WO_3_ at 1550 °C in argon resulted in the formation of W_x_C-β-SiC nanocomposite powders with significant variations in particle morphology and content of W_x_C-tipped β-SiC nano-whiskers, as revealed by TEM and SEM-EDS. The specific surface area (SSA) of the nanocomposite strongly depended on the amount of tungsten and had a notable impact on its catalytic properties for the production of furfuryl alcohol (FA) and tetrahydrofurfuryl alcohol (THFA). Nanocomposite W_x_C-β-SiC catalysts with 10 wt % W in the starting mixture had the highest SSA and the smallest W_x_C crystallites. Some 10 wt % W nanocomposite catalysts demonstrated up to 90% yield of THFA, in particular in the reduction of furfural derived from biomass, although the reproducible performance of such catalysts has yet to be achieved.

## 1. Introduction

The chemical industry is on the constant lookout for new, renewable sources of multi-purpose platform chemicals. The transformation of biomass has attracted particular interest, and numerous studies have been devoted to this subject. Biomass consists mainly of carbohydrates, which after chemical transformation can result in various products with interesting properties. Acidic hydrolysis of waste biomass can lead to the formation of furfural. Because it contains a carbonyl group and a furan ring which are reactive, this aldehyde is commonly used as a substrate in the synthesis of many desirable compounds. The catalytic reduction of furfural can produce furfuryl alcohol (FA) and both chemicals can be used further to yield tetrahydrofurfuryl alcohol (THFA) [1,2,3].

Tetrahydrofurfuryl alcohol is present in nature, in lavender and mango oils and in fermented soya. Tetrahydrofurfuryl alcohol is colorless and well miscible with water, besides which it has a high boiling point and mild smell. It is used as a green (EPA approved) solvent or as an active ingredient in many industrial products, such as cleaning liquids [4], printing inks and paints, as well as in agriculture for insecticides, pesticides, fungicides, herbicides, biocides and preparates for the protection and treatment of plants exposed to frost [5]. Agricultural THFA applications are particularly important for environmental protection, because this compound is readily biodegradable, has low toxicity and possesses excellent penetration ability. Tetrahydrofurfuryl alcohol is also widely used as an intermediate in the chemical and pharmaceutical industries. It has been tested successfully as a fuel additive [6], enabling the mixing of ethanol and diesel to produce cleaner fuel. The largest producer of THFA is “Koatsu Chemical Industries” (Osaka, Japan), which produces around 30 tons of this compound every year [1].

One method for the production of THFA is the non-selective reduction of furfural in vapor state, above 105 °C, over nickel catalysts with hydrogen gas at atmospheric pressure. However, the product obtained in this way does not have sufficient chemical purity, because it contains undesirable products of hydrogenolysis. Furthermore, the furfural poisons the surface of nickel catalysts and prevents the adsorption of hydrogen on the surface, leading to decreasing rates of furfural reduction [7]. An efficient method for obtaining THFA over nickel-chromium catalysts in the liquid phase has been patented [8]. However, this process requires a temperature above 110 °C and hydrogen pressure exceeding 100 atm. Moreover, systems containing chromium are extremely hazardous for the environment. Previous studies have shown that supported Pd, Ru, Rh and Ni catalysts modified with a second metal, such as Cu, Bi or Te, can operate selectively towards either furfuryl alcohol (FA) or THFA [9,10]. Chen et al. [11] found that the incorporation of small amounts of Pd into supported nickel systems increases THFA yield. Bimetallic systems also enable the reaction to proceed under mild conditions. However, the cost of the catalysts is higher.

Supported nickel catalysts are normally used for the production of tetrahydrofurfuryl alcohol from furfuryl alcohol [12,13,14,15]. Huiji et al. [16] found that Raney Ni catalyst has good selectivity and activity for hydrogenation of FA to THFA. However, this catalyst is not safe for use on an industrial scale, because of the demanding reaction conditions and special storage requirements. Chenguang et al. [17] investigated hydrogenation of FA over Ni and Al oxides doped with transition metals as catalysts. Processes involving such catalytic systems require high temperatures and the use of high hydrogen pressures. In order to eliminate such drawbacks, copper may be used as an additive [18]. Merat et al. [19] found that effective reduction of FA to THFA is possible using supported Ni, Pd, Ru and Rh catalysts enriched with Cu. Tike et al. [20] studied Ru/TiO_2_ systems for the reduction of FA to THFA. Seemuth et al. [21] have described a process of FA hydrogenation to THFA over zeolite catalysts containing ruthenium cations. Reduction of FA to THFA has also been performed on Pd black and Pt black powders [22]. The use of precious metal catalysts enables the reaction to proceed under milder conditions, but such systems are expensive and susceptible to poisoning.

Many other catalyst systems for the reduction of furfural to FA or THFA have been investigated. These include copper chromites [23,24], Raney metal catalysts Ni [25], Co [26] and Cu [26], nickel amorphous alloy [27,28], mixed copper-zinc oxides doped with Al, Mn or Fe [29], Cu supported on SiO_2_ [30,31], MgO [32] or carbon [33], heterogeneous and homogeneous Ru, Rh Pd or Pt catalysts [9,34,35]. Alkali (Ca, Li) or transition metals (Co, La) are incorporated into these catalysts as promoters [36,37,38]. Selectivity to FA and THFA has been shown to depend strongly on the introduction of a metallic promoter, which is explained by the different bonding strength of the furan ring on the catalyst surface [31].

It should also be remembered that when furfural is used as a substrate it is typically reduced to FA in the first step, and then other catalysts are used for further reduction of FA to THFA. The amount of THFA obtained relative to the amount of furfural used is not usually very high, because of the losses incurred during each process. As a result, the two-step process leads to a more expensive final product. Moreover, the use of chromium catalysts is hazardous to the environment, while, the use of precious metals adds to the economic costs. For these reasons, there is considerable interest in finding new types of catalyst able to transform furfural into FA or THFA, which do not use chromium or precious metals.

One-step liquid-phase catalytic processes enable low-cost production on an industrial scale, thanks to reduced energy and gas consumption. Catalysts based on noble metals (Pt, Pd, Rh, Ir, etc.) are replaced with less expensive systems, based on oxides of transition metals, metal carbides and others. Metal carbides, such as tungsten carbide (WC), have been considered as potential hydrogenation catalysts due to their electronic properties and electrical conductivities [39]. Like Pt, WC can also adsorb H_2_ [40].

In this study, nanostructured W_x_C-β-SiC composite catalysts with various amounts of W_x_C were formed by carbothermal reduction of a WO_3_ and SiO_2_ mixture in Ar atmosphere at 1150 °C. The structure and texture of the nanocomposite catalysts were analyzed using XRD, SEM-EDS, TEM, BET and FTIR. The catalytic properties of the nanostructured W_x_C-β-SiC systems were investigated for the reduction of commercial furfural to FA and THFA in aqueous phase. Experiments were also performed with furfural obtained via acidic hydrolysis of different types of biomass.

## 2. Results and Discussion

Tungsten carbide has been tested previously for the catalytic synthesis of H_2_O from H_2_ and O_2_ at ambient temperature, for the hydrogenation of WO_3_ by H_2_ in water and for 2,2-dimethylpropane isomerization into 2-methylbutane [41]. It has also been considered as a replacement for Ir systems in satellite thrusters powered by hydrazine [42]. Pd enhanced WC catalyst was used successfully in heterogeneous methane combustion [43]. Composite materials contain Pd(0) and W_2_C particles well-dispersed on carbon were investigated as hydrogen evolution catalysis [44]. Recently, there have also been reports in the literature on the catalytic activity of SiC in the one-step ethanolysis of lignin into small-molecular aromatic hydrocarbons [45]. On this basis, systems containing W_x_C have clear potential to be used as catalysts in the process of furfural reduction.

**Figure 1:**
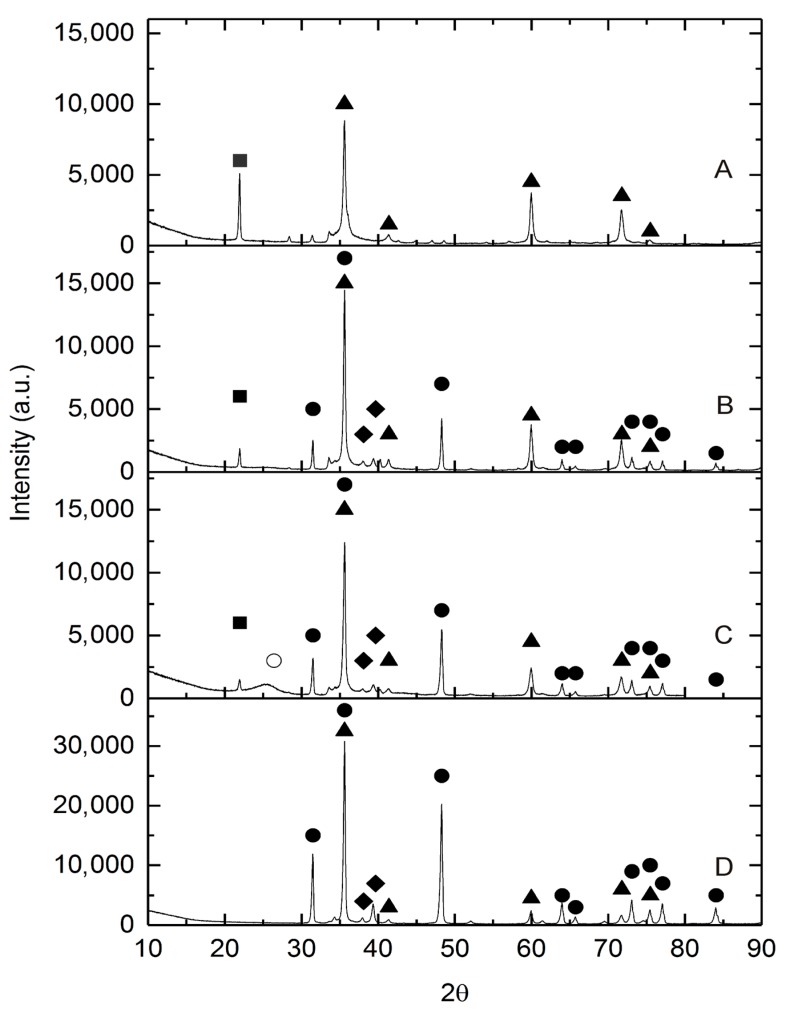
The XRD patterns of nanocomposite samples: (**A**) β-SiC; (**B**) _(5)_W_x_C-β-SiC; (**C**) _(10)_W_x_C-β-SiC and (**D**) _(20)_W_x_C-β-SiC. Crystalline phases: ▲—β-SiC, ●—WC, ◆—W_2_C, ■ cristobalite, ○ amorphous carbon. (The numbers in round brackets (5), (10) and (20) show the amounts of W in the catalysts).

In the present study, nanocomposite catalysts were prepared by carbothermal reduction of SiO_2_ or mixtures of WO_3_ and SiO_2_, and their catalytic properties were investigated for the reduction of commercial furfural in aqueous solution with the use of hydrogen gas. The results of the studies are presented in Table 1. An attempt was made to relate the catalytic properties of the nanocomposite systems to the composition of the catalyst phase (Table 2, Figure 1) and to the texture of the tested systems (Table 3).

The reaction conditions, including time of reaction, H_2_ pressure, temperature of the reaction mixture and weight of the catalyst, were optimized on the basis of previously performed experiments using the model commercial catalyst 5% Pd/Al_2_O_3_ (761176 ALDRICH). This catalyst is recommended for use in reduction reactions in aqueous phase and is well characterized by the manufacturer. The concentration of furfural in the reaction mixture was selected as 0.1 M based on experimental data obtained from acidic hydrolysis of biomass. Water solutions containing the bio-components were used in the reaction of catalytic furfural over the new nanocomposite catalysts. The results of these studies are presented in a separate subsection (Section 2.1). The results of the catalytic reduction of commercial furfural over prepared nanocomposite catalysts are shown in Table 1. The catalytic activities of the nanocomposite systems were determined by the degree of conversion to furfural [X%]. The quantities of the products of furfural reduction are shown as yields of FA [Y_FA_%] and THFA [Y_THFA_%]. These values provide a good indication of the quantities of products actually obtained from the reaction relative to the quantities of products that could theoretically have been formed from the amount of furfural amount had it reacted in its entirety. The degree of conversion to furfural [X%] and yields of FA [Y_FA_%] and THFA [Y_THFA_%] were calculated according to the formula given in Section 3.2, based on the concentrations of these chemicals in the reaction solution determined by GC-FID and GC-MS.

Both pure β-SiC and W_x_C-β-SiC nanocomposites were found to be active catalysts in the reduction of furfural, but only the _(10)_W_x_C-β-SiC system containing crystalline phases β-SiC, SiC, WC, W_2_C, SiO_2_ worked selectively towards THFA (Table 1). The addition of 5–10 wt % of W as WO_3_ to the SiO_2_ and C_graphitized_ precursor mixture led to the formation of a W_x_C-β-SiC nanocomposite with a larger surface area and smaller pore radius in comparison with the pure β-SiC system. The surface area of the _(10)_W_x_C-β-SiC nanocomposite was around six times larger than that of the pure β-SiC system (Table 3). For this catalyst, XRD measurements revealed that the WC crystallites were almost half the size of those in other W_x_C-β-SiC nanocomposites (Table 2). The combination of sufficient dispersion of the WC phase and the textural properties of the _(10)_W_x_C-β-SiC catalyst is probably responsible for the particularly high THFA yields (Y_THFA_ > 90%, Table 1) from the furfural reduction process.

Despite the high conversion of furfural over β-SiC catalyst (X = 100%, Table 1), its selectivity was not sufficient for the total reduction of all of the double bonds in furfural (Y_THFA_ = 43.2%). Furfural hydrogenation leads to the formation of a mixture of FA and THFA. However, only when both the β-SiC and SiO_2_ crystalline phases in the β-SiC catalysts were particularly high was conversion of furfural observed. The β-SiC(A) systems required double the time for annealing at high temperature and only β-SiC crystalline phase was detected by XRD (Table 2). Lower furfural conversion was observed at the end of 2 h of hydrogenation (X = 71.9%, Table 1). The specific surface area of the β-SiC(A) catalyst was the same as that of the β-SiC catalyst (Table 3), and the distribution of the products in the reaction mixture was similar.

The addition of tungsten to the β-SiC system did not influence its activity, but the yield of each product was quite different. As the W content in the W_x_C-β-SiC catalysts increased, the THFA yield rose to over 90% for the _(10)_W_x_C-β-SiC system. However, adding larger amounts of WO_3_ to the starting mixture used in the preparation of the nanocomposite materials did not lead to further improvements in the catalytic properties of the system. The _(20)_W_x_C-β-SiC catalyst, which contained 20 wt % of W as WO_3_ in the starting mixture, was less active and was not able to reduce the double bonds in the furan ring. Thus, the addition of more than 10 wt % of W to the starting mixture, which was then subjected to annealing in an inert atmosphere, does not seem justified.

The crystalline phases present in the selected catalysts were determined by XRD. The diffraction patterns are presented in Figure 1. The β-SiC sample (Figure 1A) obtained by thermal reduction of SiO_2_ with Carbon Black Vulcan XC72 at 1550 °C in argon for 90 s shows peaks at 35.8° (111), 41.51° (200), 60.21° (220), 71.91° (311) and 75.81° (222), which correspond to β-SiC phase (JCPDS Card No. 29-1129). In the X-ray diffractograms, an additional peak can be observed for cristobalite (the phase of crystalline SiO_2_) at 21.9° (101) (JCPDS Card No. 39-1425). Similarly, the XRD patterns of the composites _(5)_W_x_C-β-SiC (Figure 1B), _(10)_W_x_C-β-SiC (Figure 1C) and _(20)_W_x_C-β-SiC (Figure 1D), prepared by the thermal reduction of SiO_2_ and Carbon Black Vulcan XC72 mixture with WO_3_ additive, all show peaks characteristic of β-SiC. However, the peak intensity of cristobalite reduced with increasing amounts of W in the original mixture. Formation of W_2_C and WC phases was observed in these samples. The peaks at 31.51° (001), 48.30° (101), 64.02° (110), 73.11° (111), 77.10° (102) and 84.07° (201) for W_x_C-β-SiC-type nanocomposites may correspond with the phase of WC (JCPDS Card No. 51-0939). Characteristic peaks at 38.10° (002) and 39.67° (101) probably indicate the presence of W_2_C phase in the samples. The low intensity wide peak located at around 2θ = 26° in the XRD pattern for the (10) W_x_C-β-SiC sample (Figure 1C) can be attributed to amorphous carbon. The presence of amorphous carbon phase in only one tested sample may indicate that it is only an impurity, introduced during sample preparation, or may show a lack of homogeneity in the sample.

Crystallite size was determined using Scherrer’s equation, with experimental and instrumental profiles approximated using the Cauchy function for the SiC (111) and WC (111) peak widths. In the calculations, only negligible lattice strain was assumed (Table 2).

The specific surface areas and textural properties of the catalysts used in the reduction of furfural in an aqueous phase under hydrogen pressure, including β-SiC and W_x_C-β-SiC with different amounts of W, are summarized in Table 3. The values for furfural conversion and selectivities to individual products are listed in Table 3, as the arithmetic average of three measurements taken under the same conditions and for one batch of test catalyst.

Given the possible catalytic effects of the addition of W on the W_x_C-β-SiC composite microstructure, SEM-EDS, TEM and FTIR analyses were used to achieve a better understanding of the structure and behavior of the W_x_C-β-SiC nanocomposite catalysts and to elucidate the nature of the W_x_C and SiC species involved in the liquid phase hydrogenation of furfural.

**Figure 2:**
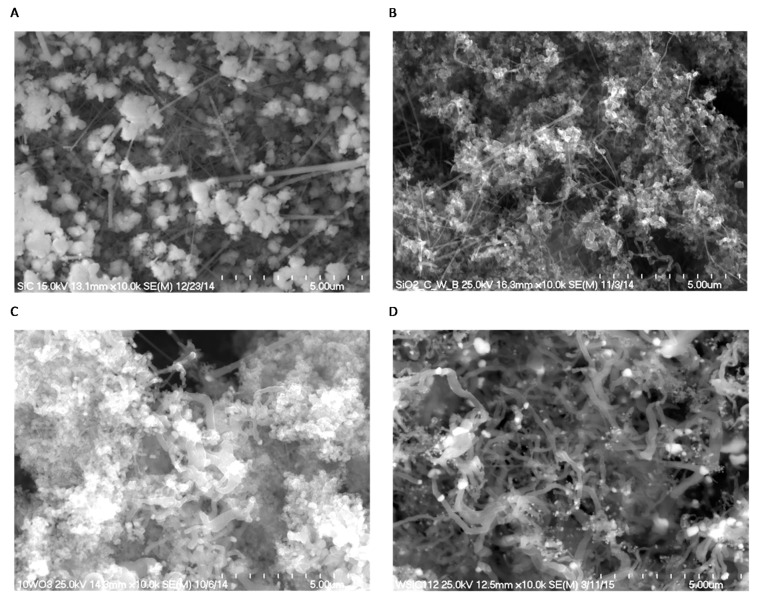
Low magnification SEM micrographs of the nanocomposites: (**A**) β-SiC; (**B**) _(5)_W_x_C-β-SiC; (**C**) _(10)_W_x_C-β-SiC and (**D**) _(20)_W_x_C-β-SiC. (The numbers in round brackets (5), (10) and (20) show the amounts of W in the catalysts).

Figure 2A shows a SEM micrograph of a pure β-SiC nanocomposite obtained by thermal reduction of SiO_2_ by graphitized carbon in an argon atmosphere. The presence of two kinds of β-SiC fibrous whiskers of different lengths and diameters in a range from 40 to 120 nm, as well as β-SiC granular nanostructures, can be observed in the sample. Significantly different morphologies were revealed for the nanocomposites formed from the C, SiO_2_ and WO_3_ mixture (Figure 2B–D).

As well as straight whiskers and granular particles of β-SiC, another β-SiC nanostructure was formed with characteristic bends along its length. The diameters of these curved nanostructures varied from 80 to 400 nm and their lengths extended to several micrometers.

It is well known from the literature data that obtaining similar nanostructures can be achieved by doping SiC catalysts with different metals or compounds [46,47,48,49,50,51,52,53,54,55,56]. The formation of SiC nano-whiskers was observed in many cases during the metal-catalyzed carbothermal reduction of SiO_2_ in mixtures with FeCl_2_ [46], Fe [47,48,49,50] and cerium oxide [51]. Both Fe and Ni have been shown to catalyze the formation of SiC nano-whiskers by the reduction of a mixture of SiO_2_ and Si with propane [52] or methane [53]. Ni and NiO have been shown to catalyze the formation of SiC nano-whiskers from NiSi systems [54]. ZnS is also a good catalyst for the formation of SiC nano-whiskers through the reaction of graphite powder mixture on Si or SiO_2_ and WO_3_ [55,56].

**Figure 3:**
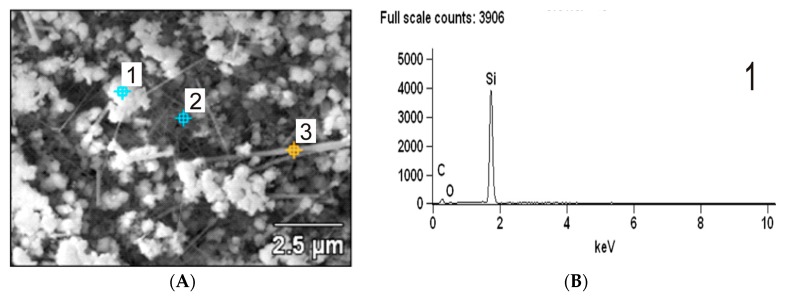
SEM/EDS analysis of the β-SiC nanocomposite obtained by thermal reduction of SiO_2_ by graphitized carbon at 1550 °C for 1.5 h (**A**) SEM image, (**B**) EDS analysis of granular structure (point 1).

To better understand the chemical composition of the nanostructures in the β-SiC nanocomposites, EDS analysis of selected micro-systems was performed. EDS analysis revealed, that the three areas showed different morphologies (granular structure (point 1); nano-whiskers structure (point 2) and nano-wire structure (point 3) Figure 3A), but the same chemical composition and a small variation of carbon.

**Figure 4:**
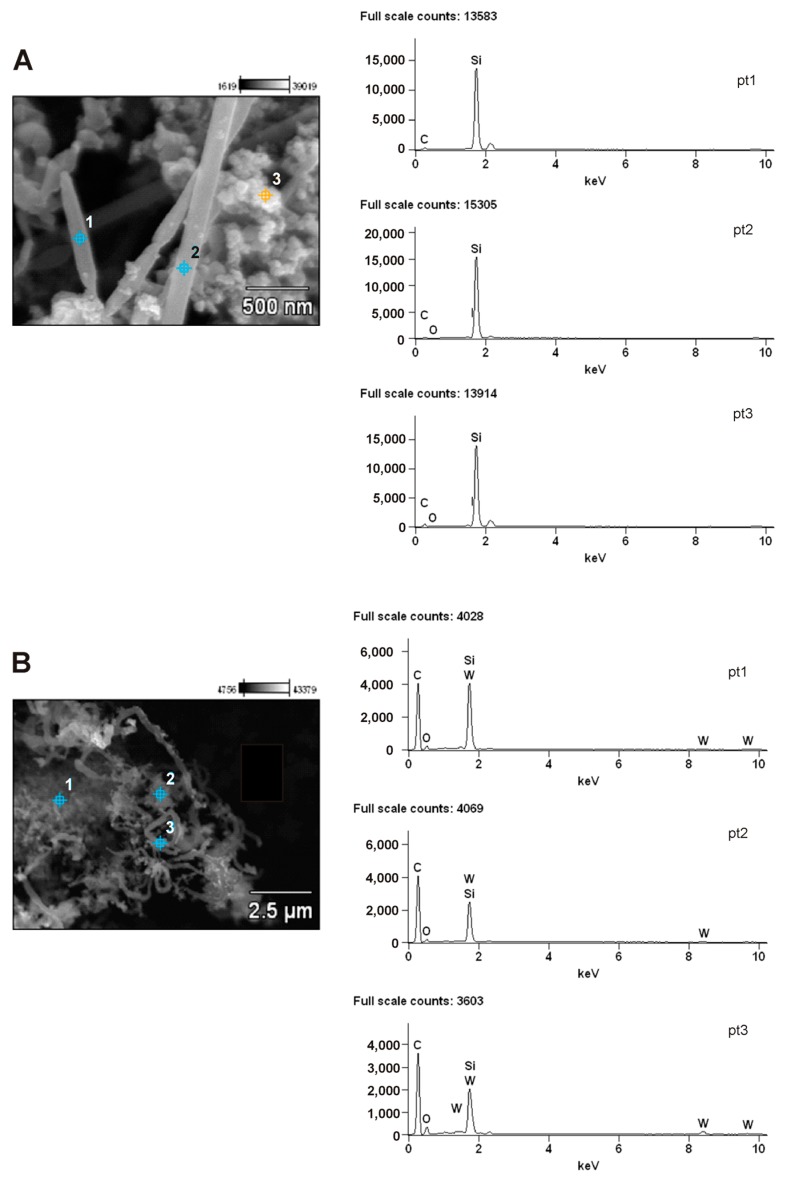
SEM/EDS analysis of the W_x_C-β-SiC nanocomposites obtained by thermal reduction of a mixture of WO_3_ and SiO_2_ by graphitized carbon at 1550 °C for 1.5 h: (**A**) SEM and EDS spectra of _(5)_W_x_C-β-SiC catalyst and (**B**) SEM and EDS spectra of _(10)_W_x_C-β-SiC catalyst. (The numbers in round brackets (5) and (10) show the amounts of W in the catalysts).

**Figure 5:**
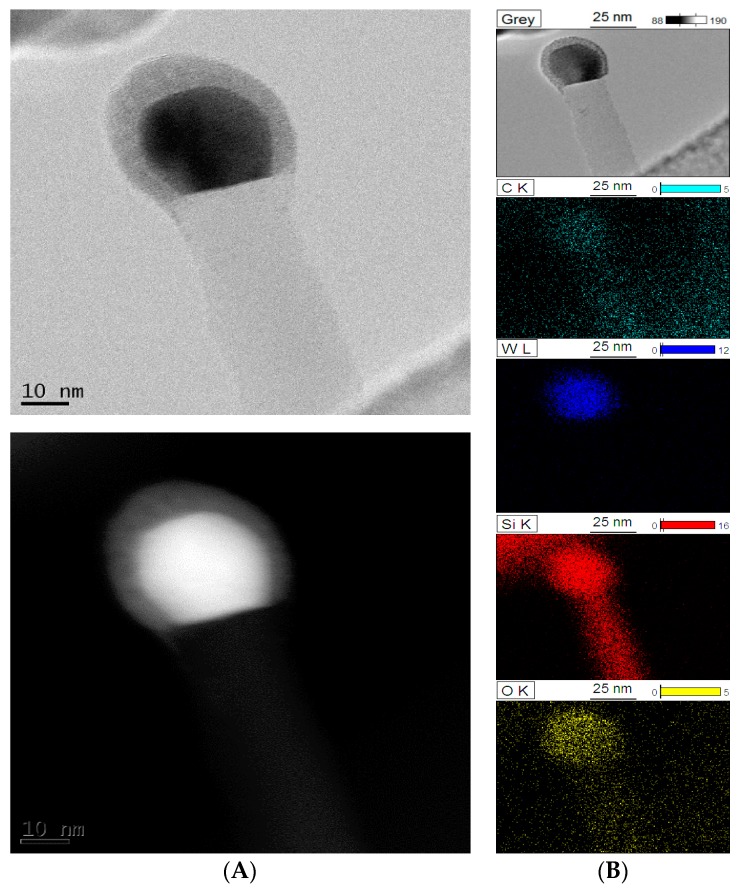
STEM images of a whisker in _(10)_W_x_C-β-SiC catalyst: (**A**) BF-STEM image and BF-STEM image with contrast; (**B**) BF-TEM image and EDS element maps of the whisker.

EDS analysis of the _(5)_W_x_C-β-SiC sample revealed the presence of tungsten homogeneously distributed in the whiskers, but this element was absent from the granular structure (Figure 4A). Increased tungsten content was observed at the tips of the straight whiskers of the _(5)_W_x_C-β-SiC nanocomposite (Figure 5). This indicates that the whiskers may have been produced via the vapor-liquid-solid (VLS) mechanism, whereas the liquid W-Si-O-C droplets formed in the reaction mixture and acted as a catalyst for the formation of SiC nano-whiskers [57,58,59,60,61,62,63,64,65,66]. The mechanisms of whisker formation have been discussed in an earlier publication [23].

The addition of larger amounts of WO_3_ (10–20 wt %) to the starting mixture of SiO_2_ and C led to the formation of rather thicker whiskers in comparison with the _(5)_W_x_C-β-SiC system (Figure 2C,D and Figure 4B). The incorporation of WO_3_ into the starting mixture of SiO_2_ and C led to the formation of W_x_C-β-SiC, which was characterized by a larger specific surface area in comparison to pure β-SiC (Table 2). This effect can be explained by the efficient formation of β-SiC and W_x_C nanostructures in the W_x_C-β-SiC composite, as opposed to the aggregates of globular particles observed in the SEM images of the β-SiC sample. However, the small amount of residual carbon in the W_x_C-β-SiC composite may also have contributed to the increase in the SSA of the W_x_C-β-SiC composite. The residual carbon was not removed from the W_x_C-β-SiC samples by annealing in air, because this process might have caused WC oxidation to WO_3_.

**Figure 6:**
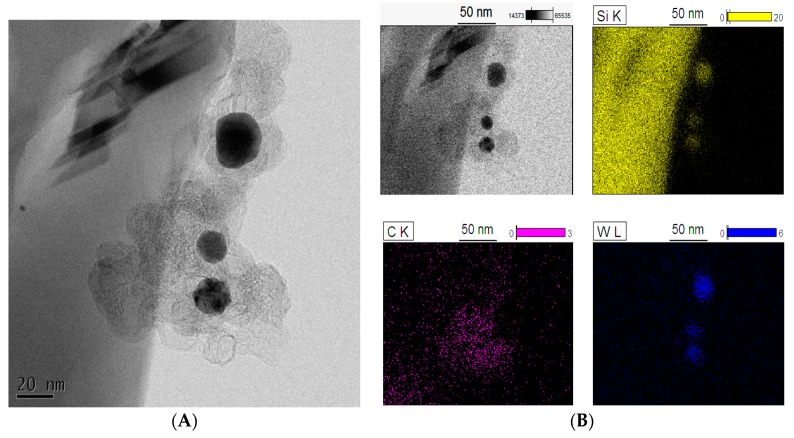
BF-TEM image (**A**) and EDS element maps (**B**) of _(10)_W_x_C-β-SiC composite.

Figure 6 shows a BF-TEM image of the _(10)_W_x_C-β-SiC nanocomposite. The W_x_C nanocrystals form what appears to be an amorphous carbon matrix on the surface of the β-SiC nanostructures. The W_x_C crystallites are distributed relatively evenly on the β-SiC nano-whiskers. Yet, a strong Si signal can also be seen in the EDS maps of the particles. Moreover, based on the BF-TEM image, the W_x_C crystallite size can be estimated as between 15 and 40 nm, which is consistent with XRD measurements (Table 1).

TEM investigations also indicated that W_x_C crystallites frequently occurred at the top of β-SiC nano-whiskers (Figure 5). A strong oxygen signal was from these areas was observed in EDS maps, perhaps due to the formation of a SiO_2_ shell around the WC core.

**Figure 7:**
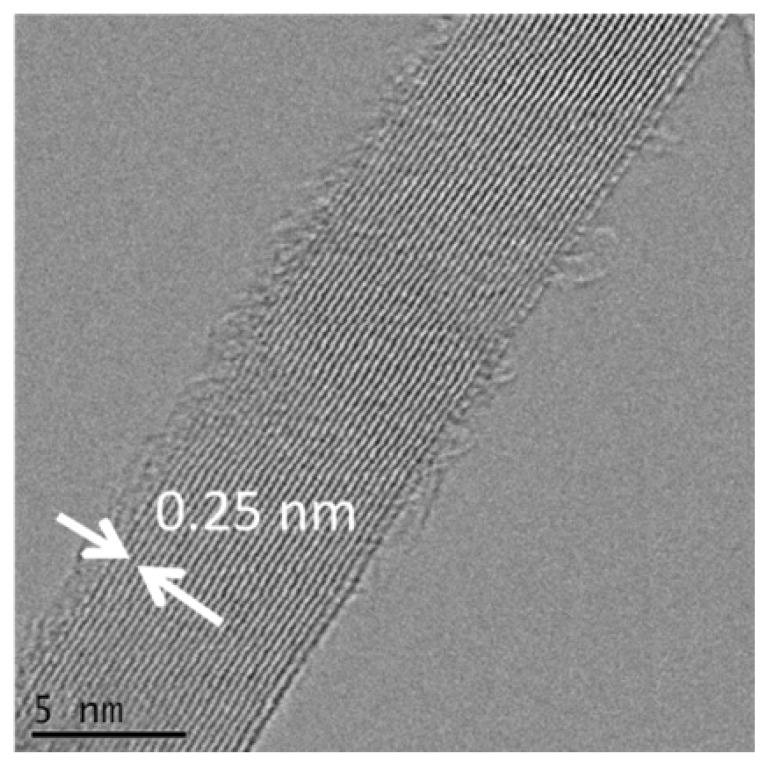
High Resolution STEM image of the whisker stem.

Figure 7 shows an HRSTEM image of the crystallite at the boundary area of the whiskers. A well-defined fringe separation of 0.25 nm can be seen, which corresponds to the lattice spacing of the (111) plane of β-SiC (JCPDS card No: 29-1129).

**Figure 8:**
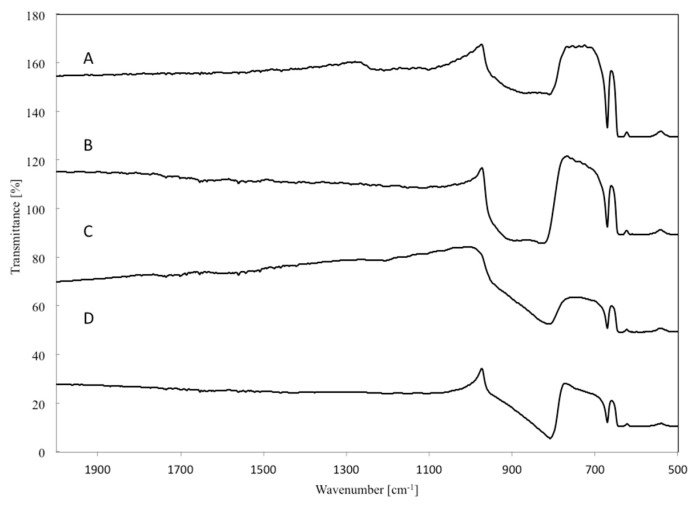
FTIR spectra for (A) β-SiC; (B) _(5)_W_x_C-β-SiC; (C) _(10)_W_x_C-β-SiC and (D) _(20)_W_x_C-β-SiC catalyst. (The numbers in round brackets (5), (10) and (20) show the amounts of W in the catalysts).

The chemical composition of the W_x_C-β-SiC catalyst was further analyzed using Fourier transform infrared spectroscopy (FTIR) (Figure 8). Figure 8A presents the FTIR spectrum in the range 500–4000 cm^−1^ for the pure β-SiC catalyst sample after annealing in argon at 1550 °C for 90 min. The FTIR spectrum shows an intense broad IR absorption band in the range from around 780 cm^−1^ to approximately 980 cm^−1^ and centered at 866 cm^−1^. This can be attributed to Si-C stretching vibrations in the β-SiC nanoparticles [57,58,59,60,61,62]. The band also shows a shift relative to the peak in Si-C stretching vibrations in bulk β-SiC (794 cm^−1^) [60,61]. A peak due to stretching vibrations in the Si–O bonds was also observed in the range from 1090 to 1100 cm^−1^, characteristic of SiO_2_ [59,63,65,66]. These observations confirm the presence of SiO_2_, which was not completely reduced by C in the annealing process.

As in the case of pure β-SiC catalyst, FTIR spectra for W_x_C-β-SiC catalysts containing 5–20 wt % of WO_3_ in the starting mixture were recorded in the range of 500–4000 cm^−1^. The corresponding FTIR spectra of the investigated nanocomposites _(5)_W_x_C-β-SiC (Figure 8B), _(10)_W_x_C-β-SiC (Figure 8C) and _(20)_W_x_C-β-SiC (Figure 8D) showed an intense IR absorption band centered at 804 cm^−1^, which can be attributed to the Si-C vibrational stretching band. The position of this band maximum differed from that observed for β-SiC obtained by the carbothermal reduction of SiO_2_ without tungsten doping. Possible reasons for this band shift may be the variations in the SiC crystallite sizes in each sample (Table 1), the formation of an amorphous tungsten oxide layer at the interface between the WC and the SiO_2_ shell (stretching O-W-O vibrations) and Si-C bond deformation at the crystallite surface in SiC nanostructures with different morphologies in β-SiC and W_x_C-β-SiC. Moreover, the FTIR spectra in Figure 8 B–D show that the addition of W into the system resulted in the complete removal of SiO_2_ from the samples, as is evident from the absence of its characteristic absorption band in the range from 1090 to 1100 cm^−1^.

### 2.1 Catalytical Transformation of Waste Biomass to THFA

Due to its ability to be used not only as a commercial product in its own right but also and primarily as a substrate in many important chemical processes, furfural is the industry’s preferred product of acid hydrolysis from biomass. Single-step conversion into THFA using the proposed nanocomposite catalysts could offer significant advantages. A number of preliminary experiments were performed with the _(10)_W_x_C-β-SiC catalyst, which had shown the best selectivity for THFA. Flax straw and oat straw as well as sugar beet pulp and sugar beet leaves were used as the waste biomass for the furfural production. In order to obtain an aqueous condensate containing bio-furfural, a portion of the biomass, equivalent to 25 g of dry matter, was placed in a 1 L round bottomed flask. For each of the biomass types used in the study, pre-determined dry matter was determined using a moisture analyzer. Next, 33 mL of H_2_SO_4_ (95%, P.P.H. “Stanlab” Sp. J.) and 92 mL of deionized H_2_O were added to the biomass and the distillation process was started. The process was terminated when the vapor temperature exceeded 100 °C. The condensate was then neutralized with Na_2_CO_3_ (POCh Gliwice SA) to pH = 7 and analyzed by HPLC, GC-FID and GC-MS to determine its qualitative and quantitative composition. Catalytic tests over _(10)_W_x_C-β-SiC catalyst were performed on the condensates (Table 4). The results were compared with those for the commercial product.

From the results presented in Table 4, it can be concluded that the nanocomposite _(10)_W_x_C-β-SiC catalyst showed particularly high activity in the process of furfural reduction. The hydrogenation of an aqueous solution of pure furfural (COMMERCIAL, POCh Gliwice S.A.) led to the formation of tetrahydrofurfuryl alcohol (THFA) as the main product. Similar results were achieved with furfural solutions obtained by the acidic hydrolysis of waste biomass. It can be concluded that the presence of trace amounts of additional compounds in the reaction mixture (acetone, 2-butanone) did not influence the activity and selectivity of the nanocomposite to individual reaction products. Waste biomass can thus be considered as a viable substrate for the production of green solvents such as THFA using nanocomposite W_x_C-β-SiC catalysts.

## 3. Materials and Methods 

### 3.1. Preparation of the Catalysts

Graphitized carbon (Carbon Black Vulcan XC72, average particle size 50 nm, Cabot Co., Boston, MA, USA) and silica (Kieselgel 60, 15–40 μm particle size, Merck KGaA, Darmstadt, Germany) powders were used as the precursors for silicon carbide formation via carbothermal reduction. The powders were mixed in a weight ratio of 1.5:1.0 silica to carbon, and the mixture was homogenized in a ball mill at 350 rpm for 30 min using zirconia balls. The homogenized mixture was loaded in a graphite furnace (VSL10/18 Degussa Wolfgang, Hanau, Germany), ramped at 30 °C·min^−1^ heating rate to 1550 °C under Ar gas flow (1 L·min^−1^), kept at 1550 °C for 90 min and then cooled naturally.

To prepare the W_x_C-β-SiC composites, WO_3_ powder (Fluka, purity 99.9%, particle size below 150 nm) was added to the silica/carbon mixture in amounts to attain 5, 10 or 20 wt % of tungsten in the final compound. The mixing and carbothermal reduction procedures were as outlined above. 

### 3.2. Hydrogenation of Furfural

Hydrogenation of furfural in aqueous solution (0.1 M·L^−1^, 25 mL) was carried out in a 50 mL stirring autoclave (Parr Instrument Company, Moline, IL, USA) at a temperature of 90 °C and under 2 MPa of H_2_ pressure. Each experiment was performed with the same load of β-SiC or W_x_C-β-SiC catalyst (*m*_cat_ = 0.5 g). The mixtures were stirred at 500 rpm. The rotational speed of the stirrer was selected on the basis of preliminary tests, so that the reaction would take place in the kinetic area. The autoclave was first flushed with Ar gas (Linde 5.0, flowrate 20 mL·min^−1^ at 20 °C for 15 min) to remove the air, and then flushed again with H_2_ gas (Air Products, Premium Plus, 99.999%, at 20 °C for 15 min). Next, the autoclave was pressurized with hydrogen to 2 MPa pressure, and the temperature was increased to 90 °C at 20 °C·min^−1^. The total reaction time was 2 h. These reaction conditions used had been reported as being optimal for mono- and bimetallic Pd catalysts [9], and enabled comparison with the β-SiC and W_x_C-β-SiC catalyst systems.

Upon completion of the reaction, the autoclave was gradually cooled to room temperature using a controlled temperature water bath. The reacted mixture was extracted, filtered and analyzed using High Performance Liquid Chromatography (LaChrome, Merck-Hitachi, Darmstadt, Germany) Kromasil 100 C_18_ column, mobile phase: acetonitrile/phosphate buffer = 5:95 *v/v*, (pH = 4.5, C_phosphate_ = 0.01, UV: λ = 210 nm) to determine the concentration of furfural. The products of furfural hydrogenation were analyzed by GC-FID (5890A, Hewlett Packard, Wilmington, DE, USA; operating parameters: packed column 8% Carbowax 1540 on Chromosorb W; injection volume: 5 µL; injection port, FID detector and column oven temperatures: 170 °C, 250 °C and 190 °C, respectively; He carrier gas (Linde, 99.999% 30 sccm flowrate)). The liquid products were also analyzed using a GC-MS analyzer (Clarus 580 with MS Clarus SQ 8 S, PerkinElmer, Waltham, MA, USA) equipped with an Elite-5MS capillary column (length 30 m, inner diameter 0.25 mm, film thickness 0.5 μm). The GC-MS analyzer operated under the following conditions: Electron Impact at 70 eV; 35–350 *m*/*z* mass range; injection port and interface temperatures 250 °C and 300 °C, respectively; column oven temperature profile: 35 °C for 7 min, ramped to 155 °C at 3 °C·min^−1^, ramped to 300 °C at 20 °C·min^−1^, and 3 min hold time at 300 °C; He carrier gas (30 sccm flowrate); 1 μL injection volume; 1:200 split ratio. 

The activity of the W_x_C-β-SiC-type nanocomposite catalysts was calculated as the percentage of furfural conversion, in accordance with the following formula:

*X* = [1 − (*C*/*C*_0_)] × 100% (1)

where *X* is the degree of the furfural conversion (%), *C*_0_ is the initial concentration of furfural (mol·L^−1^) and *C* is the furfural concentration at time *t* (mol·L^−1^).

The yield of each product from the reduction of furfural (FA, THFA, other products) was determined using the equation:

*Y* = [*C*_P_/*C*_0_)] × 100% (2)

where *C*_P_ is the concentration of the product (FA, THFA, etc.) (mol·L^−1^).

### 3.3. Analysis by X-ray Diffraction (XRD)

A PANalytical X’Pert Pro MPD diffractometer (Almelo, The Netherlands) (Cu Kα tube operated at 40 kV and 45 mA, PANalytical X’Celerator detector, Bragg–Brentano geometry) was employed to obtain X-ray diffraction (XRD) patterns. The data were acquired in the range 5–90° 2θ using a step of 0.0167° and dwelling time of 27 s. The samples were rotated during data acquisition to minimize the possibility of preferred orientation effects. Phase identification and grain size analysis were performed using the PANalytical High Score Plus software package, version: 2.3, ICDD powder diffraction file (PDF-2); International Centre for Diffraction Data (ICDD), USA, 2009.

### 3.4. Low-Temperature N_2_ Adsorption/Desorption Measurements

The textural characteristics of the β-SiC and W_x_C-β-SiC powders were determined using an automatic physisorption analyzer (ASAP 2020, Micromeritics, Norcross, GA, USA). The samples were placed in a quartz ampoule and degassed in a vacuum at 300 °C for 4 h before the measurements were taken. Specific surface area (SSA) analysis was based on the Brunauer, Emmett, Teller (BET) model for N_2_ low temperature adsorption. Barrett-Joyner-Halenda (BJH) analysis was used for volume and size analysis of pores with radii between 0.85 nm and 150.00 nm. 

### 3.5. Fourier Transform Infrared (FTIR) Measurements

Infrared transmission spectra were recorded in the range of 4000–700 cm^−1^ using a FTIR spectrometer (IRTracer-100, Shimadzu, Columbia, MD, USA) equipped with a liquid nitrogen-cooled MCT detector. The FTIR samples were prepared by mixing synthesized β-SiC and W_x_C-β-SiC powders with KBr in a ratio of 1:300 by weight. The mixtures were homogenized, dried and pressed into pellets. Pure KBr pellets were used to record and subtract the background spectra for each measurement. FTIR spectra were recorded with a resolution of 4.0 cm^−1^. A total of 128 FTIR scans per sample were taken to ensure a satisfactory signal-to-noise ratio.

### 3.6. Scanning Electron Microscopy (SEM) Analysis of β-SiC and W_x_C-β-SiC

A scanning electron microscope (SEM S-4700, Hitachi, Tokyo, Japan) equipped with an energy dispersive spectrometer (EDS, Thermo Noran Inc., Madison, WI, USA) was used to analyze the size, shape, surface morphology and elemental composition of the synthesized β-SiC and W_x_C-β-SiC powders. All observations were performed with 25 kV accelerating voltage. Both secondary electron (SE) and back-scattered electron (BSE) images were acquired at several magnifications for better visualization of specific micro-regions of interest in the samples. The elemental composition of different micro-regions with distinct morphological features was analyzed using Energy Dispersive Spectroscopy (EDS) (Thermo Noran Inc.).

### 3.7. Transmission Electron Microscopy (TEM) Analysis

Transmission Electron Microscopy (TEM) analysis of the synthesized β-SiC and W_x_C-β-SiC powders was carried out using a scanning-transmission electron microscope (STEM HD2700, Hitachi) equipped with an EDS system and operated at a 200 kV accelerating voltage. The powder particles were precipitated on a holey carbon film on Cu 300 mesh TEM-grids. EDS elemental maps of selected micro-regions were obtained and used for phase identification and to determine the elemental composition of the nanocomposite particles. 

## 4. Conclusions

From this investigation into the aqueous phase hydrogenation of furfural in the presence of nanocomposite W_x_C-β-SiC catalysts, the following conclusions can be made:

(a) In the case of pure β-SiC catalysts, the activity of the systems depends on the presence of both β-SiC and SiO_2_ crystalline phases. This means that incomplete carbothermal reduction of SiO_2_ leads to the development of the system surface and improves its activity. However, the reduction of furfural is not selective and the process produces similar yields of FA and THFA.
(b) While the addition of tungsten does not influence furfural conversion by β-SiC systems, it does affect the type of products produced in the reaction mixture. As the amount of tungsten in the β-SiC system was increased up to 10% by weight, the THFA yields also rose. The best selectivity to THFA was achieved with nanocomposite _(10)_W_x_C-β-SiC catalyst. The improved selectivity of _(10)_W_x_C-β-SiC catalyst to THFA is probably attributable to the sufficient dispersion of WC phase in this nanocomposite, in combination with its textural properties. The addition of larger amounts of W into the catalyst did not lead to further improvement of selectivity for THFA.
(c) The results obtained by SEM, TEM and FTIR techniques reveal that the addition of tungsten into the β-SiC system facilitates the formation of whiskers and reduction of SiO_2_ by C in the carbothermal reduction process.
(d) The nanocomposite _(10)_W_x_C-β-SiC catalyst was used successfully for the reduction of an aqueous solution of furfural obtained by acid hydrolysis of flax and oat straws. Despite the presence of trace amounts of organic impurities in the substratum obtained from the biomass, the _(10)_W_x_C-β-SiC catalyst was characterized by a very high activity and selectively for THFA. After optimization of the process conditions, this two-step catalytic process (I—acidic hydrolysis of biomass; II—catalytic reduction of Furfural to THFA) has potential to be used for the commercial production of green solvent from waste biomass.

## Figures and Tables

**Table 1:** Conversion of furfural and yields of FA and THFA after the 2 h hydrogenation of furfural over β-SiC and W_x_C-β-SiC catalysts. The results in the table are expressed as the arithmetic mean of a minimum of three measurements.

Catalyst	Furfural Conversion (%)		Yield (%)	
		FA	THFA	Other Products *
β-SiC	100	54.8	43.2	0
_(5)_W_x_C-β-SiC	100	49.7	48.9	1.3
_(10)_W_x_C-β-SiC	100	8.4	90.5	1.1
_(20)_W_x_C-β-SiC	67.6	42.2	24.6	0.3
_(10)_W_x_C-β-SiC(A)	100	53.2	46.8	0.4
_(10)_W_x_C-β-SiC(B)	69.4	58.1	11.3	0.1
_(10)_W_x_C-β-SiC(C)	35.1	29.6	5.9	0.2
β-SiC(A)	71.9	53.9	18.0	0.5
β-SiC(B)	73.4	60.7	12.7	0.3

* Other products: THF, MTHF, products of condensation of FA (mainly 5-fufuryl-furfuryl alcohol, but also 2,2′-difurylmethane, 2-(2-furylmethyl)-5-methylfuran, difurfuryl ether, 4-furfuryl-2-pentenoic acid γ-lactone, 2,5-difurfurylfuran and 2,2′-(furylmethylene)bis(5-methylfuran)). Conditions of the reaction: C_furfural_ = 0.1 M, m_cat_ = 0.5 g, T = 90 °C, V_furfural_ = 25 mL, p_H_2__ = 20 bar. (The numbers in round brackets (5), (10) and (20) show the amounts of W in the catalysts).

**Table 2:** Average crystallite size in WxC-β-SiC-type nanocomposites.

Catalyst	Temp. of Carbothermal Reduction (°C)	Time of Carbothermal Reduction (h)	Crystalline Phases	Size of SiC Crystallites (nm)	Size of WC Crystallites (nm)
β-SiC,	1550	1.5	β-SiC, SiO_2_	36	-
_(5)_W_x_C-β-SiC	1550	1.5	β-SiC,SiC, WC, W_2_C, SiO_2_	37	50
_(10)_W_x_C-β-SiC	1550	1.5	β-SiC,SiC, WC, W_2_C, SiO_2_	23	32
_(20)_W_x_C-β-SiC	1550	1.5	β-SiC,SiC, WC, W_2_C, SiO_2_	18	47
_(10)_W_x_C-β-SiC(A)	1550	3.0	β-SiC,SiC, WC, W_2_C	35	37
_(10)_W_x_C-β-SiC(B)	1550	4.5	β-SiC,SiC, WC, W_2_C	37	42
_(10)_W_x_C-β-SiC(C)	1550	1.5	β-SiC,SiC, WC, W_2_C	25	23
β-SiC(A)	1550	3.0	β-SiC	36	-
β-SiC(B)	1550	1.5	β-SiC	20	-

(The numbers in round brackets (5), (10) and (20) show the amounts of W in the catalysts).

**Table 3:** Specific surface areas and textural properties of the catalysts used in the experiments.

Catalyst	W wt % in the Starting Mixture	Surface Area (m^2^/g)	Total Pore Volume (cm^3^/g)	Average Pore Radius (nm)
β-SiC	0	9.6	0.034	8.59
_(5)_W_x_C-β-SiC	5	45.4	0.145	6.17
_(10)_W_x_C-β-SiC	10	57.4	0.184	6.10
_(20)_W_x_C-β-SiC	20	10.0	0.035	6.67
_(10)_W_x_C-β-SiC(A)	10	29.2	-	-
_(10)_W_x_C-β-SiC(B)	10	24.9	-	-
_(10)_W_x_C-β-SiC(C)	10	31.0	-	-
β-SiC(A)	0	9.6	-	-
β-SiC(B)	0	11.2	-	-

(The numbers in round brackets (5), (10) and (20) show the amounts of W in the catalysts).

**Table 4:** Catalytic properties of _(10)_W_x_C-β-SiC catalysts after 2 h of bio-furfural reduction. The results in the table are expressed as the arithmetic mean of a minimum of three measurements.

Origin of Furfural	Furfural Concentration (mol·L^−1^)	Conversion of Furfural (%)	Yield (%)		
			FA	THFA	Other Products *
COMMERCIAL, POCh Gliwice S.A.	0.10	100	8.4	90.5	1.1
Acidic hydrolysis of oat straw	0.11	100	8.6	89.9	1.5
Acidic hydrolysis of flax straw	0.08	100	7.1	92.3	0.6
Acidic hydrolysis of sugar beet pulp	0.06	100	9.1	89.8	1.1
Acidic hydrolysis of sugar beet leaves	0.02	100	10.5	86.2	3.3

* Other products: THF, MTHF, products of condensation of FA (mainly 5-fufuryl-furfuryl alcohol, but also 2,2′-difurylmethane, 2-(2-furylmethyl)-5-methylfuran, difurfuryl ether, 4-furfuryl-2-pentenoic acid γ-lactone, 2,5-difurfurylfuran and 2,2′-(furylmethylene)bis(5-methylfuran)). Conditions of the reaction: C_furfural_ = 0.1 M, m_cat_ = 0.5 g, T = 90 °C, V_furfural_ = 25 mL, p_H_2__ = 20 bar.
